# Group 2 Vaccinia Virus, Brazil

**DOI:** 10.3201/eid1812.120145

**Published:** 2012-12

**Authors:** Felipe Lopes Assis, Iara Apolinario Borges, Paulo César Peregrino Ferreira, Cláudio Antônio Bonjardim, Giliane de Souza Trindade, Zélia Inês Portela Lobato, Maria Isabel Maldonado Guedes, Vaz Mesquita, Erna Geessien Kroon, Jônatas Santos Abrahão

**Affiliations:** Author affiliation: Universidade Federal de Minas Gerais, Belo Horizonte, Minas Gerais, Brazil

**Keywords:** poxvirus, Orthopoxvirus, vaccinia virus, outbreak, viruses, cattle, Brazil

## Abstract

In 2011, vaccinia virus caused an outbreak of bovine vaccinia, affecting dairy cattle and dairy workers in Brazil. Genetic and phenotypic analyses identified this isolate as distinct from others recently identified, thereby reinforcing the hypothesis that different vaccinia virus strains co-circulate in Brazil.

Throughout most of Brazil, vaccinia virus (VACV), family *Poxviridae*, is the etiologic agent of bovine vaccinia (1). Outbreaks often occur on unhygienic rural properties and cause mild to severe rashes on teats and udders of dairy cows and various locations on humans (1,2). Dairy workers usually seek medical care for the painful lesions, but rarely are they hospitalized. Some studies suggest an association between these outbreaks of bovine vaccinia and the VACV strains used during the World Health Organization smallpox eradication campaign (3). Since 1999, VACV strains in Brazil have been investigated (2–8); biological and molecular approaches indicated 2 distinct groups of these viruses (9,10). In 2011, a bovine vaccinia outbreak occurred in Serro County, Minas Gerais state, in southeastern Brazil, one of the largest milk-producing regions in Brazil. The outbreak affected 91 dairy cows and 3 dairy workers, 1 of whom was hospitalized (Technical Appendix Figure 1, panel A). Our aim was to elucidate the genetic and phenotypic characteristics of this VACV isolate.

## The Study

**Figure 1:**
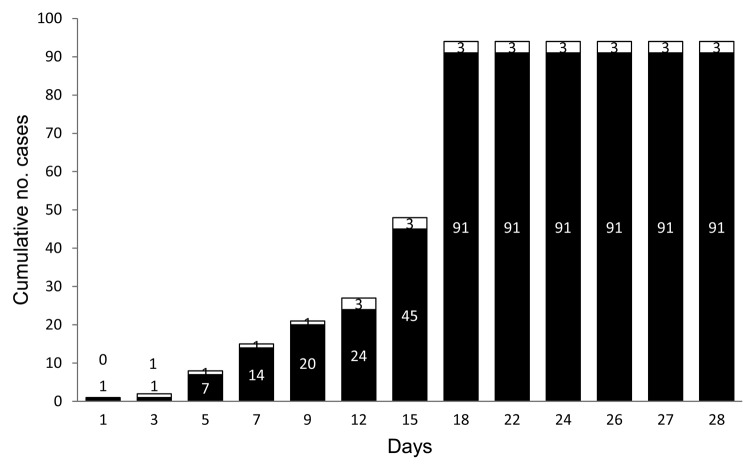
Timeline of 2011 vaccinia virus (VACV) outbreak in Serro County, Minas Gerais state, southeastern Brazil, involving 2 neighboring farms, 91 dairy cattle, and 3 dairy farm workers. Day 1, first case of bovine vaccinia in cow, farm 1; day 3, first case of human infection, patient 1, farm 1; day 5, more cases in cattle, farm 1; day 7, entire herd of cattle sick, farm 1; day 9, first 6 cows sick, farm 2; day 12, second and third human cases (patients B and C); day 15, patient C hospitalized; day 18, entire herd of cattle sick, farm 2; day 22, cumulative (both farms) mild production decrease of 70%; day 24, both farms quarantined; day 26, patient C returns to work, with lesions; day 27, lesion samples collected from patients B and C and 1 cow, farm 2; day 28, all cattle recovering.

The outbreak affected 2 farms, 91 cows, and 3 humans (Figure 1). On day 1, on farm 1, the index case was a sick cow with ulcerative lesions on the teats and udder. On day 3, the owner of farm 1 (patient A), who had had direct contact with the sick cow, noticed lesions on his hand. On day 5, another 6 cows on farm 1 became sick, and patient A went to farm 2 and handled healthy cattle. On day 7, all 14 cows on farm 1 were sick. On day 9, some cows on farm 2 became sick. On day 12, the owner of farm 2 (patient B) and his employee (patient C) became sick. On day 15, patient C was hospitalized with high fever, lymphadenopathy, prostration, and painful vesicular–pustular lesions on his hands and arms. He received clinical support and remained hospitalized for 10 days. He had no immunologic disorders and took no medications that could be associated with his severe clinical condition. According our investigation, only those 3 patients had direct contact with the infected cattle during the outbreak.

By day 18, all 77 cows on farm 2 were sick. On day 24, veterinary surveillance teams isolated these farms for 8 days. On day 26, patient C returned to work although lesions remained on his hands and arms (Technical Appendix Figure 1, panel B). By day 28, all cattle were recovering. 

To identify the etiologic agent responsible for the outbreak, on day 27 we collected swab samples from lesions of patients B and C (not from patient A, whose lesions were healing) and from 1 infected cow (from farm 2). Samples were placed in Vero cells for virus isolation as described (2) and then purified in a sucrose gradient (11). The isolates from the patients B and C and the cow were named VACV Serro human 1/2011 (SH1V/2011), VACV Serro human 2/2011 (SH2V/2011), and VACV Serro bovine 1/2011 (SB1V/2011), respectively. 

**Figure 2:**
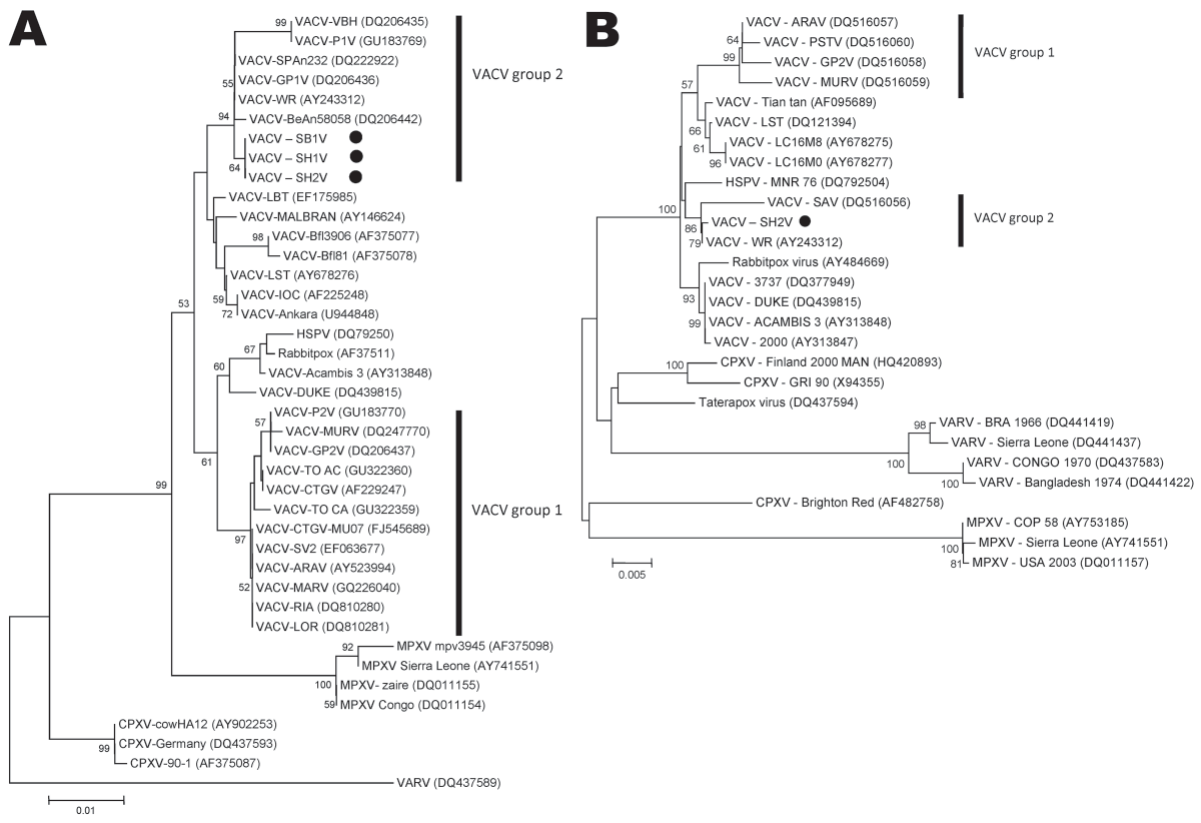
Phylogenetic analysis of vaccinia virus (VACV) isolates. A) Phylogenetic tree based on the nucleotide sequence of the orthopoxvirus hemagglutinin gene. VACV Serro bovine (SB1V), VACV Serro human 1 and 2 (SH1V and SH2V) grouped with VACV group 2 isolates, far from other VACV group 1 members. These isolates grouped far from (outliers) Serro-2 virus, a VACV isolated in the same geographic region. B) Phylogenetic tree based on the nucleotide sequence of the orthopoxvirus *ati* gene. The hemagglutinin tree shows SH2V grouping with VACV Western Reserve (WR) and SPAn232 virus, members of VACV group 2, not close to other isolates from Brazil. The neighbor-joining method with the Tamura-Nei model of nucleotide substitutions in the MEGA4 software program (www.megasoftware.net/*)* was used. Bootstrap confidence intervals are shown on branches (1,000 replicates) with GenBank accession numbers. MPXV, monkeypox virus; CPXV, cowpox virus; HSPV, horsepox virus; VARV, variola virus. Black dots indicate samples isolated during the 2011 outbreak of bovine vaccinia in Brazil. Scale bars indicate nucleotide substitutions per site.

The isolates were examined by PCR for the *A56R* gene (hemagglutinin [HA]), and the fragments obtained (950 bp) were sequenced and analyzed as described (2,12,13). The nucleotide sequences showed 100% identity among all VACV Serro 2011 isolates and exhibited high identity with VACV strains from Brazil, particularly SPAn232 (99.8% identity) and GuaraniP1 (99.5% identity) viruses. The HA phylogenetic tree clustered most VACV isolates from Brazil together, mainly because of the presence of the deletion signature (group 1). However, VACV Serro 2011 isolates did not exhibit this signature and instead clustered with VACV strains that are less frequently isolated during outbreaks in Brazil (group 2) (Figure 2, panel A).

To further characterize the virus, we also sequenced the *A26L* gene (*ati*) (14). Because HA sequences were identical for SH1V/2011, SH2V/2011, and SB1V/2011 (and hypothetically represent the same isolate), we selected SH2V/2011 to analyze for *ati* and virulence in BALB/c mice. The *ati* gene is highly polymorphic in VACV strains from Brazil, and some strains exhibit a large deletion in the *ati* gene (15). Therefore, we used *ati* to characterize the VACV strains from Brazil. A 1,600-bp fragment that also did not contain the deletion was amplified from SH2V/2011. This virus exhibits no deletion in either the HA or *ati* genes in the analyzed regions of these genes. The *ati* phylogenetic tree (Figure 2, panel B) showed that SH2V/2011 also clustered with SPAn232 virus (group 2) and was segregated from the other group 1 VACV strains.

Given the atypical genetic profile of SH2V/2011 and the long-term hospitalization of patient C, we investigated the virulence of this isolate in mice (following the rules of Committee of Ethics for Animal Experimentation, Universidade Federal de Minas Gerais, Belo Horizonte, Minas Gerais, Brazil). A total of 16 BALB/c mice were divided into 4 groups of 4 mice each. We intranasally inoculated 4 mice with 10-μL doses of viral suspensions containing 10^6^ PFUs, as described (10). Two groups were inoculated with VAVC-GuaraniP1 and VACV-GuaraniP2 as virulent and nonvirulent controls, respectively. Another group was inoculated with phosphate-buffered saline. The SH2V/2011 sample was highly virulent in BALB/c mice; morbidity rate was high (Technical Appendix Figure 2), thereby supporting the grouping of this sample in the virulent cluster. The animals that were infected with SH2V/2011 exhibited ruffled fur, arched backs, and weight loss, much like those infected with VACV-GuaraniP1. No clinical signs were observed in mice that had been inoculated with either VACV-GuaraniP2 or phosphate-buffered saline. 

To further characterize the virus, we also performed a plaque phenotype assay in BSC-40 cell cultures. The VACV strains from Brazil that exhibited virulence in a BALB/c model usually formed large plaques in BSC-40 cell cultures. This assay showed that, in contrast to VACV- GuaraniP2, in BSC-40 cell cultures, SH2V/2011 induces the formation of large plaques that are similar to those induced by VACV-Western Reserve and VACV-GuaraniP1 (Technical Appendix Figure 3).

## Conclusions

Our results indicate that VACV Serro-2011 is a new mouse-virulent VACV strain associated with an outbreak that affected cows and humans. Recently, several VACV strains have been isolated in Brazil, most exhibiting a signature deletion in *A56R*, few or no deletions in the *ati* gene, and low virulence in mouse models. In contrast, the VACV isolated from the outbreak reported here, affecting cattle and humans, exhibited virulence in mice but no deletions in either the *A56R* or *ati* genes. This strain is genetically and phenotypically distinct from the Serro-2 strain that was isolated from the same region in 2005 (7); thus, >1 VACV might be circulating in Serro and possibly other regions of Brazil (7,8).

During outbreaks of bovine vaccinia, hospitalization of humans, especially for several days, is unusual. Unfortunately, despite the development of in vivo models for study and differentiation of VACV from Brazil, no clear association between viral genetics and disease severity in humans and cattle has been shown. Although preliminary, the data presented here indicate a possible association between these 2 factors, considering the hospitalization of patient C to be long term. Although the surveillance and characterization of VACV have advanced in recent years, observation and description of any clinical characteristics of infected humans and cattle are still helpful. Such observations might be associated with genotypic and phenotypic features of VACV, which could influence surveillance and control strategies for the management of VACV outbreaks.

Technical Appendix Vaccinia virus outbreak in Serro County, Minas Gerais state, southeastern Brazil. Appendix shows location of outbreak, lesions on human patient, clinical signs of control and experimentally infected mice, and results of phenotypic plaque-forming testing of vaccinia viruses on epithelial kidney cells BSC-40.
