# Supplementary material for: Group 2 Vaccinia Virus, Brazil
Source: Emerg Infect Dis. 2012 Dec;18(12):2035–8. doi: 10.3201/eid1812.120145 (PMC3557889; doi:10.3201/eid1812.120145)
Supplement: Technical Appendix — Vaccinia virus outbreak in Serro County, Minas Gerais state, southeastern Brazil. Appendix shows location of outbreak, lesions on human patient, clinical signs of control and experimentally infected mice, and results of phenotypic plaque-forming testing of vaccinia viruses on epithelial kidney cells BSC-40. [file 12-0145-Techapp-s1.pdf]

Article DOI: http://dx.doi.org/10.3201/eid1812.120145

Group 2 Vaccinia Virus, Brazil

Technical Appendix

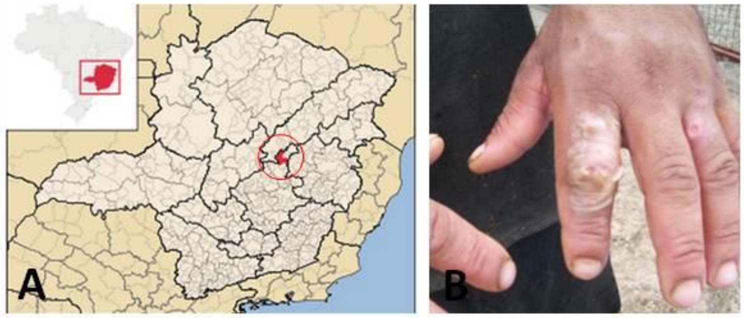

Technical Appendix Figure 1. A) Location of 2011 vaccinia virus outbreak in Serro County, a dairy production region of Minas Gerais state, southeastern Brazil. Square in inset, Minas Gerais state; circle on main map, Serro region. B) Ulcerative lesion on dairy worker’s hand (patient C).

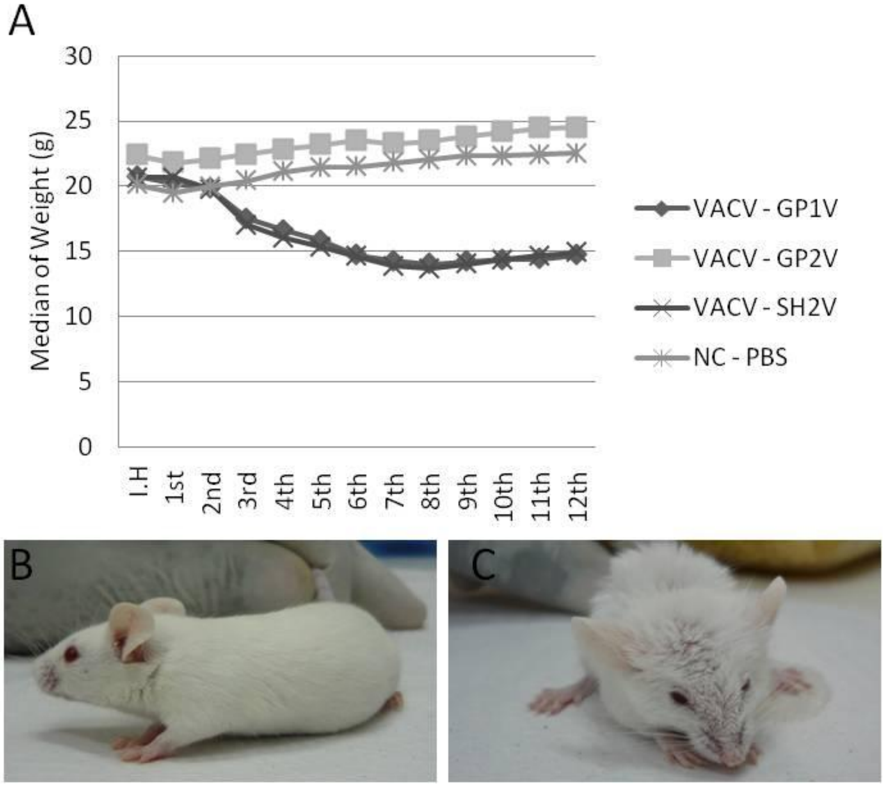

Technical Appendix Figure 2. Clinical signs of BALB/c mice infected by intranasal inoculation with10^6 PFU/10 µL vaccinia virus (VACV). A) Median weight loss relative to initial weight. Inoculation with VACV

GuaraniP1 virus (GP1V) and VACV Serro human 2/2011 (SH2V) showed similar weight loss (67.5% and 66.3% respectively). B) Control mouse inoculated with VACV GuaraniP2 (GP2V) virus. No clinical signals were observed in control mice inoculated with phosphate-buffered saline (NC-PBS) and GP2V. C) Mouse inoculated with SH2V, showing clinical signs. Severe clinical signs, such as weight loss, ruffled fur, arched back, closed eyes with inflammation, and facial edema were observed in mice inoculated with VACV GuaraniP1 and SH2V.

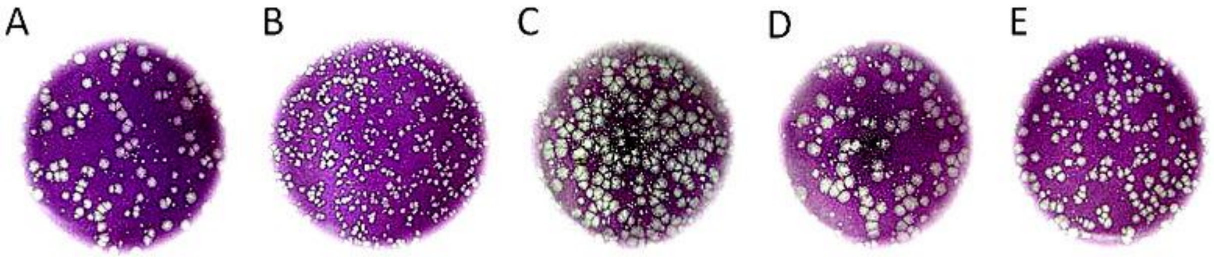

Technical Appendix Figure 3. Results of phenotypic plaque-forming testing of vaccinia viruses (VACVs) on epithelial kidney cells BSC-40. A) VACV GuaraniP1 virus; B) VACV GuaraniP2 virus; C) VACV Serro human; D) VACV Serro bovine; E) VACV Western Reserve. Viruses in panels C and D show similar large cytopathic effects, like VACV GuaraniP1 virus, members of VACV group 2, and VACV Western Reserve. VACV virus shows smaller plaques on cell culture, like other VACV group 1 viruses.
